# A new electrochemical method that mimics phosphorylation of the core tau peptide K18 enables kinetic and structural analysis of intermediates and assembly

**DOI:** 10.1016/j.jbc.2023.103011

**Published:** 2023-02-11

**Authors:** Eloise Masquelier, Esther Taxon, Sheng-Ping Liang, Yahya Al Sabeh, Lior Sepunaru, Michael J. Gordon, Daniel E. Morse

**Affiliations:** 1 Institute for Collaborative Biotechnologies, University of California, Santa Barbara, California, USA; 2 Materials Department, University of California, Santa Barbara, California, USA; 3 Department of Molecular, Cellular and Developmental Biology, University of California, Santa Barbara, California, USA; 4 Department of Chemistry and Biochemistry, University of California, Santa Barbara, California, USA; 5 Department of Chemical Engineering, University of California, Santa Barbara, California, USA

**Keywords:** tau, electrochemistry, protein, assembly, folding aggregation, fibrils, oligomers, neurodegenerative, Alzheimer’s, CE, counter electrode, DLS, dynamic light scattering, DPV, differential pulse voltammetry, E-CD, electrochemically driven CD, E-DLS, electrochemically driven DLS, EDX, energy dispersive x-ray spectroscopy, LLPS, liquid-liquid phase-separated, OCP, open circuit potential, RE, reference electrode, TEM, transmission electron microscopy, ThT, thioflavin-T, WE, working electrode

## Abstract

Tau protein’s reversible assembly and binding of microtubules in brain neurons are regulated by charge-neutralizing phosphorylation, while its hyperphosphorylation drives the irreversible formation of cytotoxic filaments associated with neurodegenerative diseases. However, the structural changes that facilitate these diverse functions are unclear. Here, we analyzed K18, a core peptide of tau, using newly developed spectroelectrochemical instrumentation that enables electroreduction as a surrogate for charge neutralization by phosphorylation, with simultaneous, real-time quantitative analyses of the resulting conformational transitions and assembly. We observed a tipping point between behaviors that paralleled the transition between tau’s physiologically required, reversible folding and assembly and the irreversibility of assemblies. The resulting rapidly electroassembled structures represent the first fibrillar tangles of K18 that have been formed *in vitro* at room temperature without using heparin or other charge-complementary anionic partners. These methods make it possible to (*i*) trigger and analyze in real time the early stages of conformational transitions and assembly without the need for preformed seeds, heterogenous coacervation, or crowding; (*ii*) kinetically resolve and potentially isolate never-before-seen early intermediates in these processes; and (*iii*) develop assays for additional factors and mechanisms that can direct the trajectory of assembly from physiologically benign and reversible to potentially pathological and irreversible structures. We anticipate wide applicability of these methods to other amyloidogenic systems and beyond.

Signal-activated, enzymatically catalyzed phosphorylation is one of the most evolutionarily ancient and widely distributed mechanisms regulating the structure and function of proteins in all kingdoms of life on earth (1). Tau, the principle microtubule-associated protein in neurons, is regulated by phosphorylation in its physiologically normal binding to microtubules (2, 3) and driven by hyperphosphorylation to form cytotoxic amyloid filaments and tangles associated with Alzheimer’s disease and other tauopathies (2, 4, 5, 6). In fact, of 38 recognized amyloid diseases associated with 20 proteins including tau, 25 (66%) are associated with phosphorylation of these proteins (7, 8, 9, 10, 11, 12). Conversely, mutations inactivating tau kinases have been found to reduce the severity (13) or delay the onset (14) of the tau amyloid-associated neurodegeneration of Alzheimer’s disease.

K18, a 126-residue peptide containing the four repeats of the tubulin-binding domain of tau (15) is driven by phosphorylation to form liquid–liquid phase-separated (LLPS) droplets (16), a state from which full-length tau further condenses to form amyloid fibrils (17, 18). K18, which like tau is strongly cationic, also can be seeded to form amyloid-like filaments by preformed tau amyloid (19, 20) and by complementary anionic polymers and compounds (21, 22) and accordingly has been used as a model for analyses of factors controlling amyloid assembly.

K18 is a cationic, block-copolymer-like peptide (15), with 20 lysine residues (ca. 16%), 5 histidines (4%), and 1 arginine (<1%), accounting for its high pI of 8.24 (23). Overlapping sets of its serine, threonine, and tyrosine residues have been found to be phosphorylated in tau isolated from the brains of normal and Alzheimer’s disease human subjects (24). We recently demonstrated that reflectin, a cationic, block-copolymeric, signal-transducing protein normally triggered by phosphorylation-mediated charge neutralization to undergo secondary folding and assembly, can be induced to undergo these transitions by pH titration (25, 26) and low-voltage electroreduction (27) acting as surrogates for the physiological, charge-neutralizing phosphorylation. K18 also is cationic, block copolymeric, and triggered to assemble by phosphorylation. These findings inspired us to investigate the possibility that low-voltage electroreduction could be used as a surrogate for phosphorylation to induce kinetically controlled folding and assembly of K18. Taking advantage of the catalytic efficiency of platinum for proton electroreduction (28, 29, 30), we show here that low-voltage electroreduction of the lysine residues in K18 can serve as a surrogate for charge neutralization by phosphorylation, driving irreversible secondary folding and hierarchical assembly to rapidly form beta-rich fibrillar tangles. In contrast to results with reflectin that exhibits dynamic arrest of assembly and reversal of assembly when charge neutralization is reversed (25, 27), K18, under specific conditions, continues to undergo progressive folding and assembly once triggered by electroreduction, resembling its known prion-like behavior in amyloid seeding (19). Perhaps most significantly, we observe a previously unsuspected metastability as a function of conditions, revealing a tipping point between divergent trajectories that parallel those of tau *in vivo*, leading either to the reversible assembly physiologically required for its interaction with tubulin or to potentially polymorphic (31, 32) and pathological amyloid formation. An advantage of this new electrochemical method over phosphorylation, heterogeneous coacervation, and other previously used techniques is its unique ability to simultaneously control, kinetically resolve, and analyze the transitions involved.

## Results

### Droplet DPV of K18

**Figure 1:**
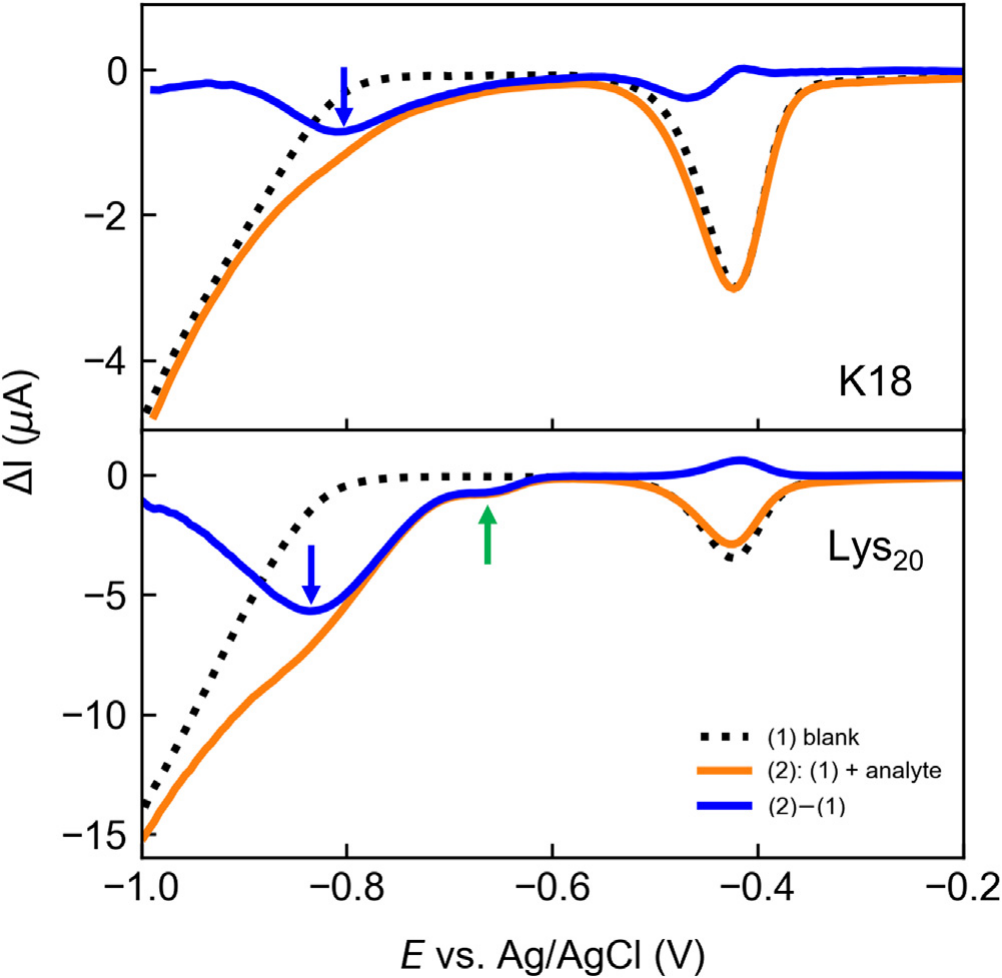
**Differential pulse voltammograms of K18 and Lys**_**20**_**show electroreduction of lysine side chains.***Blue* curves ((2)-(1)) are the deconvolved voltammograms of K18 and Lys_20_ after subtraction of the differential pulse voltammetry without analyte (*blank*, *dotted line*) from that with analyte (*orange*). *Arrows* indicate the redox feature of ε-amino side chains (*blue down arrow*) and N terminus (*green up arrow*). Dynamic light scattering analyses (not shown) confirmed that K18 was monomeric.

Examination of the sequence of K18 suggests that its cationic character is dominated by the positively charged side chains of its abundant lysine residues (comprising ca. 16% of the sequence), with a lesser contribution from histidine (4%). This suggestion is experimentally confirmed by differential pulse voltammetry (DPV) of K18, performed at pH 3 to ensure full protonation of the lysine and histidine residues of the peptide (Fig. 1), in agreement with prior results with Lys_20_ poly-L-lysine (33).

The results for K18 exhibit a clear electroreduction wave at ca. – 0.8 V, with a shoulder at – 0.7 V. By comparison with Lys_20_, which shows two distinct waves corresponding to the reduction of the ε-amino group (– 0.85 V) and the N terminus (– 0.65 V) (33), these may be assigned to the ε-amino group of lysine residues of the K18 peptide. Reduction of the imidazolium side chain of histidine could also contribute to the trailing edge of the K18 wave, considering that its redox potential has been observed at ca. – 0.6 V (27, 29).

### Electrochemical charge neutralization drives folding and assembly

**Figure 2:**
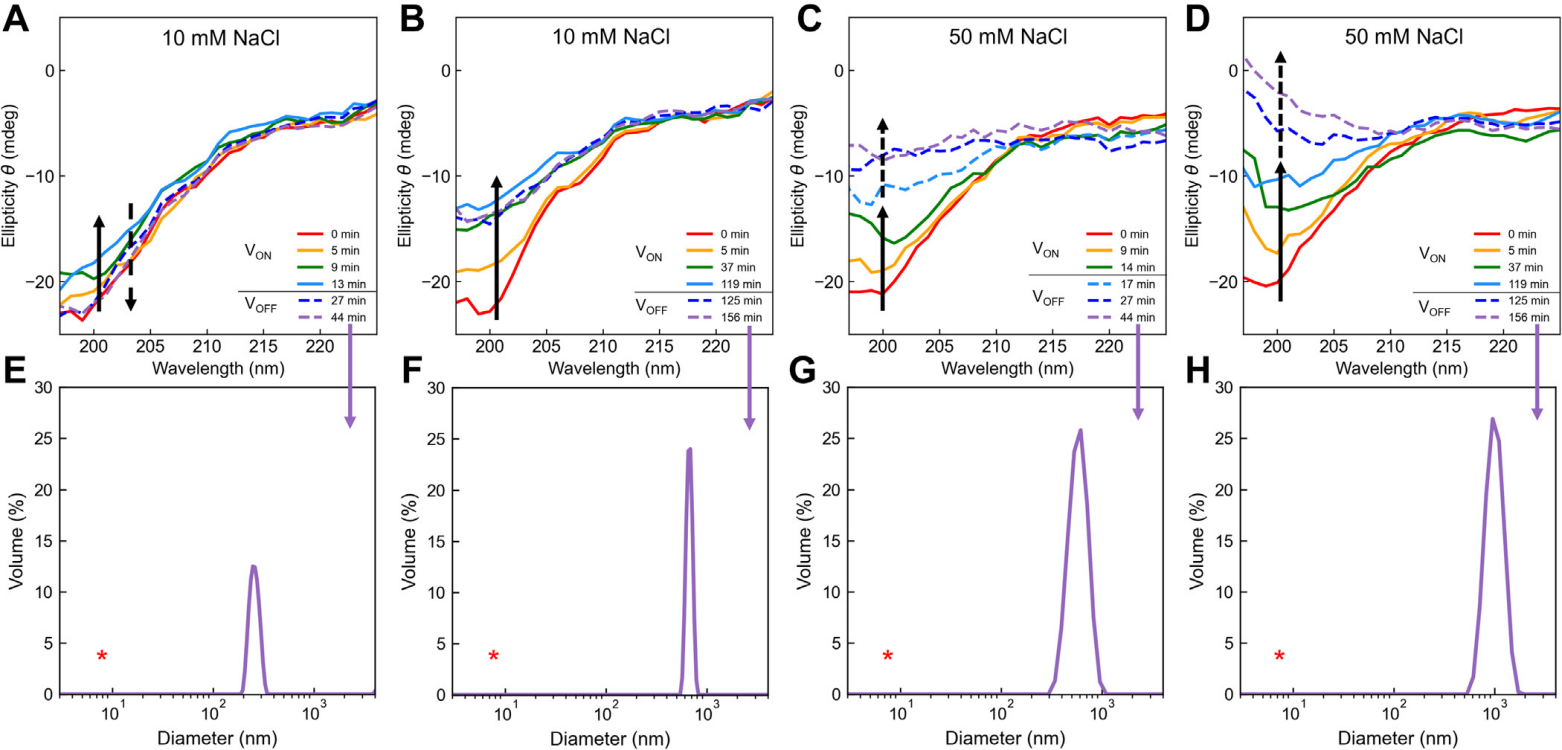
**Electroreduction of K18 drives secondary folding and assembly.** Dynamic evolution of ellipticity as a function of time in 10 mM NaCl (*A* and *B*) and 50 mM NaCl (*C* and *D*) of K18 solutions exposed to an applied potential of – 0.9 V for 15 min (*A* and *C*) and 120 min (*B* and *D*). CD is continuously monitored during this exposure and shortly after the potential is returned to open circuit potential (V_OFF_) (*solid curves, solid arrows, and dashed curves, dashed arrows*, respectively). Dynamic light scattering of K18 solutions before (indicated by *red* ∗) and after the indicated electrochemically driven CD (*E*–*H*), revealing changes from monomer to assemblies.

To evaluate the conformational response of the K18 peptide to charge neutralization resulting from the electroreduction observed in Figure 1, we continually monitored circular dichroism (CD) changes induced during and shortly following exposure to a potential of – 0.9 V at 10 mM and 50 mM NaCl (electrochemically driven CD [E-CD]; Fig. 2, *A*–*D*). Dynamic light scattering (DLS) measurements were taken before (*e.g.*, Fig. S1*B*) and after E-CD to evaluate assembly (Fig. 2, *E*–*H*). This potential was chosen to ensure electroreduction of both lysine and histidine side chains, as verified previously (27, 29, 33) and observed in Figure 1.

Using instrumentation that we described previously (33), simultaneous, real-time monitoring of ellipticity determined by CD and UV absorption driven by the applied potential revealed that exposure of K18 to – 0.9 V triggered the progressive evolution of structural changes. In 10 mM NaCl, a short exposure to the applied potential (15 min) led to a reversible loss of the random coil conformation, as observed by an increase in ellipticity at 200 nm that fully returned to its initial value when the potential was returned to open circuit potential (OCP; *i.e.*, equilibrium potential at which no current is flowing in the cell) (Fig. 2*A*). Interestingly, DLS post E-CD showed formation of 300-nm-diameter assemblies, suggesting that under this condition the short exposure to the applied potential and the resulting structural rearrangement was accompanied by the conversion of monomers to oligomers (Fig. 2*E*). Longer exposure (120 min) to the same potential drove irreversible structural change of K18 and the formation of larger (ca. 750 nm diameter) assemblies (Fig. 2, *B* and *F*). The dynamic evolution of absorbance (Fig. S1*C*) revealed an isosbestic point at ca. 205 nm, consistent with a two-state transition from the initial random coil conformation to a second state. While the exact nature of that second state cannot be unequivocally determined from CD data, the observed pattern suggests inclusion of a mixture of alpha and beta conformations (32), as previously observed for K18, tau, and their constituent peptides in the condensed state (5, 34, 35). The same experiments repeated at 50 mM NaCl showed faster and more pronounced structural change, and significantly, the continuous evolution of secondary structure after the potential was returned to OCP (Fig. 2, *C* and *D*). DLS analyses following E-CD at the higher salt concentration revealed larger assemblies with a wider distribution relative to those observed at the lower salt concentration condition. Electrochemically induced secondary folding (measured by changes in ellipticity) and assembly size (measured by DLS) thus are dependent on the salt concentration and time of exposure to – 0.9 V, showing greater changes and continued development after return to OCP at higher salt concentration and longer exposure to the applied potential. Fluorometric analysis of K18 with thioflavin-T (ThT) after E-CD in 50 mM NaCl confirmed the formation of beta-rich structures from the unstructured monomers prior to electroreduction (Fig. S3).

### Gradient of behaviors; continuation of assembly

**Figure 3:**
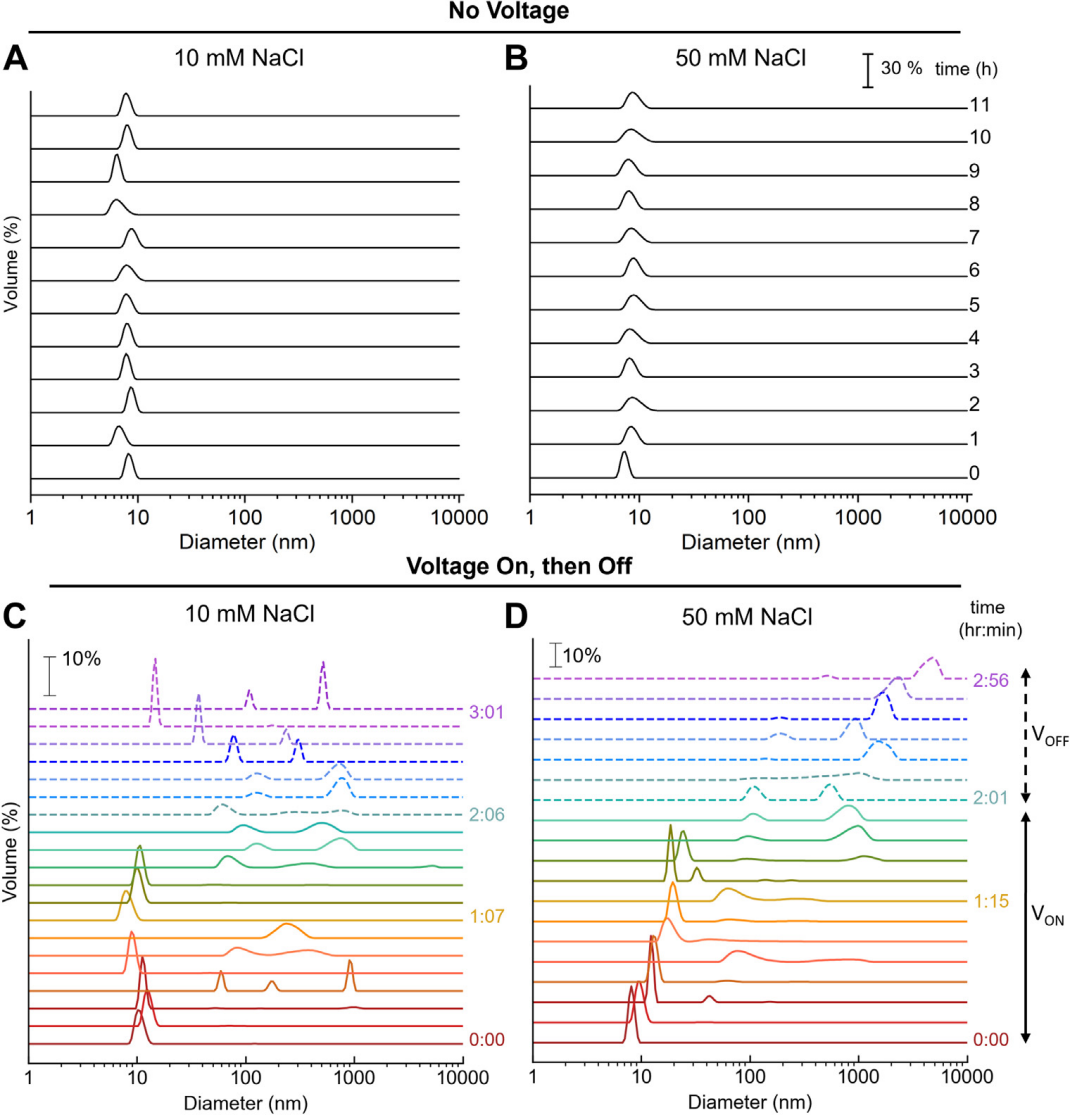
**Dynamics of K18 assembly depend on salt concentration.** Dynamic light scattering (DLS) of K18 particle size distribution at 10 mM NaCl (*A* and *C*) and 50 mM NaCl (*B* and *D*). DLS was measured with no voltage applied (*A* and *B*) for 11 h and with voltage on (V_ON_ at – 0.9 V, for 120 min) and subsequently returned to open circuit potential (V_OFF_ for 40 min) (*C* and *D*). DLS was continuously measured during and after the potential is switched off (*solid and dashed lines*, respectively).

Spectroelectrochemical analyses permitting direct, real-time measurement of electrochemically driven changes in assembly monitored by DLS (27) (Fig. 3) extend the finding of a gradient of behaviors seen in Figure 2. The K18 monomer remains stable and unassembled in the absence of an applied potential, in both 10 and 50 mM NaCl, over at least 11 h (Fig. 3, *A* and *B*). In contrast, analysis at the applied potential of –0.9 V unequivocally reveals progressive and electrical potential-, time-, and salt-dependent assembly (Fig. 3, *C* and *D*). Over the 80 min at –0.9 V in 10 mM NaCl (Fig. 3*C*), K18 exhibited pronounced metastability with a mixture of monomers (∼10 nm diameter) and transient assemblies (100–300 nm). With further time of exposure, assemblies were heterogenous and metastable, reaching ca. 2000 nm. After the potential was returned to OCP, the assembly stopped growing and monomers were intermittently observed over the next 40 min, demonstrating dynamic evolution of assemblies, followed by their partial disassembly after the return to OCP. In contrast, at 50 mM NaCl, a progressive growth was observed to begin rapidly after application of the potential, indicating the formation of larger assemblies. Significantly, these continued to grow after the return to OCP, suggestive of autocatalytic seeding, in marked contrast to the “dynamic arrest” exhibited by reflectin (25) and other LLPS-forming proteins (35, 36). These results were closely paralleled by the continued, progressive increase in count rate recorded in the DLS measurements (Fig. S2*B*), confirming the increase of assembly size. No return to monomers was observed. Interestingly, the size of the assemblies formed within the first 120 min of applied potential in both salt concentrations were of the same order of magnitude, yet those formed in 10 mM NaCl appeared and disappeared ephemerally, while continuous growth was observed in 50 mM NaCl.

### Transmission electron microscopy and energy dispersive X-ray spectroscopy of assemblies

**Figure 4:**
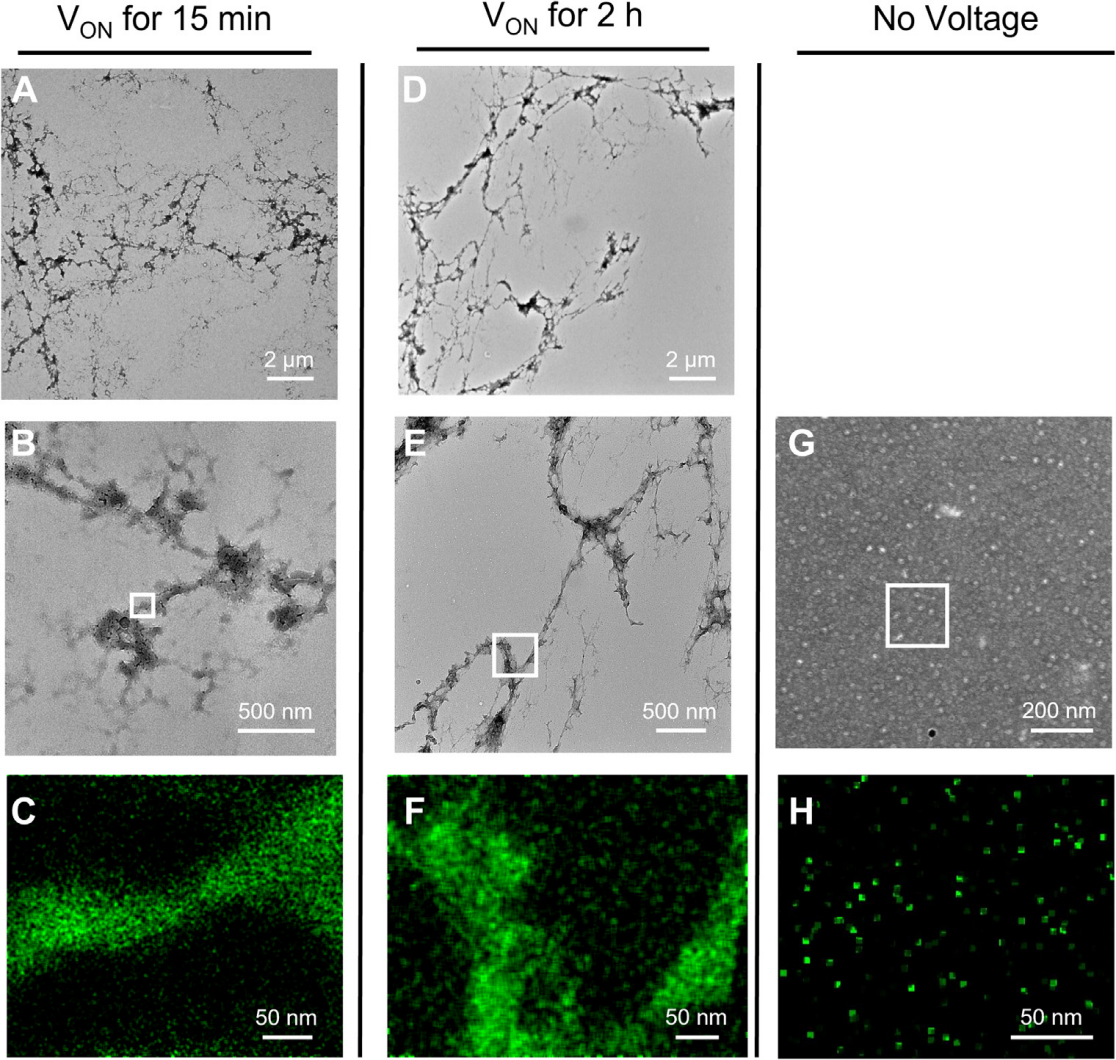
**Electroreduction rapidly drives assembly of K18 to form fibrillar tangles.** Samples were analyzed by electrochemically driven CD at 50 mM NaCl with applied voltage (V_ON_) for 15 min as described for Figure 2, withdrawn, and quickly stained on a TEM grid and analyzed by TEM (*A* and *B*). A second sample was analyzed identically by electrochemically driven CD but with voltage applied for 2 h followed by 40 min at open circuit potential and then imaged by TEM (*D* and *E*). Control analysis of K18 under conditions identical to those in (*A* and *D*) but with no applied voltage show only monomers (*G*), confirming the dependence of assembly on electroreduction. Close-up analyses for nitrogen by energy dispersive X-ray spectroscopy (*C*, *F*, and *H*) of the structures indicated by the white rectangles in (*B*, *E* and *G*), respectively, confirmed the compositions as protein. Energy dispersive X-ray spectroscopy analysis of a section of the grid containing no evident structure showed no differential nitrogen content. TEM, transmission electron microscopy.

Transmission electron microscopy (TEM) confirmed the presence of tangled, filamentous assemblies after 15 min of E-CD performed (and with results observed) as in Figure 2*C* (Fig. 4, *A* and *B*), with somewhat more tightly condensed filaments formed after 2 h of exposure to the neutralizing potential followed by another 40 min of assembly after the voltage had returned to OCP (Fig. 4, *D* and *E*). Parallel samples of K18 incubated identically, but with no applied voltage, showed only the K18 monomers, with no assemblies (Fig. 4, *G* and *H*), consistent with results in Figure 3*B* and confirming the dependence of assembly on electrochemical reduction. EDX analyses for nitrogen confirm the composition of the assemblies (Fig. 4, *C* and *F*) and monomers (Fig. 4*H*) as protein.

It is not surprising that the fibrillar networks seen in Figure 4 differ from the more regular, rigid, paired helical filaments and related structures seen on assembly of K18 with heparin sulfate and other complementary anionic polymers or heterogeneous coacervate amyloid structures of full-length tau (32, 37, 38), as assembly of K18 was driven here in the absence of such structure-directing, complementary charged polymers or the additional flanking N- and C-terminal domains of tau.

## Discussion

Electrochemistry has previously been used to measure and investigate tau and its assemblies (39, 40) and the aggregation of other amyloidogenic proteins including alpha-synuclein (41, 42) and amyloid beta (39, 43, 44, 45, 46, 47). However, the method and observations reported here are the first to simultaneously drive and quantitatively monitor these processes in real time in such a protein, using new hybrid instrumentation that combines electrochemical triggering of folding and assembly with *in situ* spectrophotometric (UV, CD, and DLS) quantitative analyses (with independent confirmation of the electroreductive formation of beta-structured filaments by TEM and fluorometric analysis with ThT). Taking advantage of the ability of low-voltage electroreduction to serve as an *in vitro* surrogate for the charge neutralization of cationic proteins resulting from phosphorylation (and hyperphosphorylation) (27, 29, 33), our results demonstrate that we can use this method to drive and kinetically control the folding and rapid assembly of tau-derived K18 to form beta-rich fibrillar tangles. In contrast to the previous demonstration that folding and assembly of reflectin, with its cationic character dominated by histidine residues (pKa = ca. 6.5), can be driven by electroreduction at ca. – 0.6 V, these processes in the lysine (pKa = ca. 10)-dominated K18 are driven by electroreduction at – 0.9 V, the potential observed to drive electroreduction (cf. Fig. 1) and conformational transition of polylysines (33). These results support the hypotheses that (i) Coulombic repulsion maintains the initial random coil conformation of the monomer of K18 (pI = 9.73) and its full-length tau parent (pI = 8.24) and (ii) charge neutralization by phosphorylation *in vivo* and electroreduction progressively overcome this repulsion to drive condensation, folding, and assembly *in vitro*, as previously observed for reflectin (25, 26, 27), suggesting that this technique may be useful to further elucidate the early committed stages, exogenous regulators, and factors selecting different trajectories of assembly in amyloidosis. We anticipate that further analyses of K18 and full-length tau performed in conjunction with structural resolution by cryo-TEM will determine (i) the degree to which the products formed by electroassembly resemble those identified in human pathologies; (ii) whether the products of electroassembly are single polymorphs, as typically found in disease-derived filaments, or multiple polymorphs as formed through coacervation with heparin (37); and (iii) whether this method may help identify additional factors governing the divergent trajectories to the specific polymorphs found in different tauopathies and their locations in the brain (31, 32).

The CD data reported in Figure 2 indicate a progressive loss of K18’s initial random coil conformation with transition to a mixture of alpha and beta structures. The observed conformational changes are irreversible upon removal of the applied potential in 50 mM NaCl, even after only a short time of exposure to the applied potential (Fig. 2*C*), indicating considerable stability. Prior NMR analyses of the K19 region of tau (including a portion of the microtubule-binding domain that is wholly present in K18) suggest that the initial formation of β-sheet within microtubule-binding repeat 3 (shared by K18 and K19) may be an important early nucleus of amyloid formation, even though only a small portion of the protein adopts this conformation (48). Furthermore, *in silico* modeling of the four individual microtubule-binding repeats of tau found that, while all repeats most frequently adopted a random conformation in the nonphosphorylated state, helices and sheets formed transiently. Repeat 3 in particular adopted a β-sheet structure 18% of the time, and this propensity rose to ∼50% when the simulation modeled a dimer (49).

*In vitro* assembly of nonmutant and nonphosphorylated full-length tau previously has required complementary inducers such as heparin or SDS (50). Relatedly, a recent analysis of hyperphosphorylated tau revealed only amorphous assemblies formed *in vitro* in the absence of complementary charge partners (6), consistent with previous work showing such partners or structural modification to be required for rigidly structured assemblies (6, 49). In contrast to the relatively slow heterogeneous coacervation forming filaments of K18 and tau typical of previous observations (16, 51, 52), beta-rich filamentous tangles of K18 are formed rapidly in response to low-voltage electrochemical reduction, in the absence of anionic partners, mutations, molecular seeding, or crowding (Figure 2, Figure 3, Figure 4). The rapidity of the electroreductively induced folding, assembly, and formation of these filamentous networks may be attributed in part to the electroreductive, catalytic efficiency of the Pt working electrode (WE), the physical mechanism of which has recently been elucidated (30). Analysis of the K18 assemblies with ThT reveals an increase in fluorescence relative to that of the monomer, confirming the presence of beta-rich fibrils (Fig. S3). Interestingly, the increase in ThT fluorescence of K18 following electroreduction appear to be significantly higher than that previously reported for K18 fibrils formed by agitation in the absence of heparin (52).

Control experiments confirmed that the K18 monomers undergo no structural change or assembly in the absence of an applied potential at both salt concentrations tested (Fig. 3, *A* and *B*). In marked contrast, application of the electrochemically reductive potential drives significant changes in folding revealed by CD (Fig. 2) and assembly revealed by DLS (Fig. 3, *C* and *D*). Comparison of the kinetics of these effects (although analyzed under slightly different conditions; cf. Methods) suggests that folding may be required for assembly; the highly cationic random coil monomer does not spontaneously form multimers (Fig. 3, *A* and *B*). Significantly, while longer exposure at – 0.9 V in 50 mM NaCl leads to the irreversible formation of larger assemblies, even a brief exposure of 15 min under this condition drives formation of some assemblies of ca. 600 nm in diameter (Fig. 2*G*). In comparison, K18 assembly triggered in the absence of aggregation inducers typically required hours or days before filaments were observed (52, 53).

Unlike results for reflectin under comparable conditions, in which cessation of the applied potential results in rapid reversal of the induced assembly (27), return of K18 to OCP results in no such reversal at 50 mM NaCl. In fact, continued time after return to OCP results in continued folding and assembly (Figs. 2, *C* and *D* and 3*B*), resembling prion-like seeding. E-CD at – 0.45 V, a potential that drives only hydronium reduction, produced no detectable changes either in K18’s ellipticity or absorbance in 10 mM NaCl (Fig. S5, *A* and *C*), but a clear transition and continuous growth was observed after the potential was returned to OCP in 50 mM NaCl (Fig. S4, *B* and *D*). In view of the fact that the pH gradients in both salt concentrations are identical at this potential (cf. the overlapping DPV traces for hydronium reduction in Fig. S5), this result can likely be attributed to the enhanced charge screening conferred by the higher salt concentration (54). In contrast, at – 0.9 V, faster kinetics of water electrolysis are seen at the higher salt concentration, leading to a stronger pH gradient. This is confirmed by observation of increased UV absorbance due to the formation of OH^-^ (55) in the no-protein E-CD control at – 0.9 V at this higher salt concentration, while no change in absorbance is detected in the parallel condition at 10 mM NaCl (Fig. S6). Interestingly, the kinetics of structural change at – 0.9 V in both salt concentrations are comparable (Fig. S7), suggesting that direct (electrode contact mediated) electroreduction of the ε-amino groups of K18’s lysine residues is the primary driver of charge neutralization of the protein. Notably, all experiments performed in 50 mM NaCl show a continuous evolution of structures and assembly after the potential is returned to OCP, in contrast to the behavior in 10 mM NaCl. We observe this difference regardless of the initial driving potential or the duration of its application (Fig. S7). We hypothesize that significant concentration gradients of Na^+^ and Cl^-^ ions exist in the diffusion layer to compensate for the strong pH gradient seen at higher salt concentration, maintaining electroneutrality. When the potential is returned to OCP, the fall of those gradients back to their bulk concentrations would suddenly create local increases in Cl^-^ ions, which could drive further folding and assembly through enhanced charge screening and hydrophobic interactions (56, 57). We conclude that the progression or gradient of behaviors observed, from (i) stable monomer to (ii) metastable, reversibly folded and assembled, and (iii) irreversible, beta-rich fibrillar tangles, is thus driven and finely tuned by the cumulative effects of electrochemically controlled, phosphomimetic charge neutralization. In addition to the effect of salt in screening the positive charges of the cationic K18 (54), the role of salt on the assembly processes of tau recombinants has been highlighted in a recent study, in which specific cations were observed to impact the shapes and density of the structures formed (50). Further exploration of the role of salt may help identify specific conditions necessary to form filaments matching disease structures.

The delayed, transitory, and partially reversible assembly of K18 observed after prolonged exposure to – 0.9 V at 10 mM NaCl, the more rapid and progressive growth of assemblies at that potential and 50 mM NaCl (Fig. 3), and the transition between reversibility and irreversibility of conformational change with length of exposure to that potential at the low-salt condition (Fig. 2, *A* and *B*), all indicate one or more charge neutralization thresholds for assembly and irreversibility. It is interesting to note that the progression or gradient of behaviors observed in Figures 2 and 3 parallels and may underlie in some fundamental way the progression *in vivo* from normal, physiologically essential, and reversible phosphorylation and consequent changes in the parent tau protein to the progressively more pathological and irreversible amyloid state. That possibility would suggest that *in vivo*, as observed *in vitro* here, this transition can, under certain conditions, be a gradual one, rather than the result of a single all-or-none switch. That suggestion is supported by the recent findings that (i) the brain of a deceased Alzheimer’s disease subject contained numerous forms of tau differing in their extent of phosphorylation, seeding ability, and resistance to protease (58) and (ii) populations of tau isolated from different Alzheimer’s disease individuals differed in their kinetics of seeding (59).

Fibril formation of tau and K18 driven by heterogeneous coacervation with complementary charged, polyanionic polymers such as heparan sulfate and RNA have been well studied (19, 20, 22, 60), but recent work has called into question the applicability of such complex coacervation to disease, as heparan sulfate–derived amyloids do not closely resemble disease amyloid (61). While such complex formation depends in large part on the positive charges of the protein’s multiple lysine residues, the simple assembly reported here is *opposed* by the Coulombic repulsion of the charged lysine residues, driven by electroreduction of the ε-amino groups of lysine residues and facilitated by charge-neutralizing pH titration and salt screening. Related to these observations, acetylations of tau in the K18 region, which have the dual effect of simultaneously decreasing positive charge and increasing hydrophobicity, have been linked to tauopathies (62, 63, 64). Similarly, a naturally occurring mutation that deletes a lysine residue at a specific position in the K18 portion of tau is amyloidogenic both *in vivo* and *in vitro* and highly correlated with a familial neurodegenerative frontotemporal dementia (65, 66). These results suggest that the electroreduction-driven conformational changes and assembly reported here may be directly relevant to those underlying neurodegenerative tauopathies.

## Conclusion

The pronounced metastability observed in K18’s structural transitions, which had been computationally predicted and experimentally and genetically analyzed for tau and its component peptide domains (48, 66), is kinetically resolved here as a result of the unique advantage of the electrochemical method used (27). The time-dependent accumulation of incremental reductions occurring with each diffusionally limited contact of protein with the electrode enables the identification of, and experimental and analytical accessibility to, a tipping point between the divergent trajectories that parallel those of tau *in vivo*, leading either to the reversible assembly physiologically required for its interaction with tubulin or to potentially pathological amyloid formation. Specific advantages of the methods described, through their induction of low-voltage, time- and salt concentration-dependent, include (i) the ability to control and analyze in real time, and potentially isolate, never-before-seen intermediates in the process of folding and assembly; (ii) the ability to conduct such analyzes under conditions yielding either more physiologically normal or pathological outcomes; (iii) the ability to conduct such analyses without the need for preformed seeds, heterogeneous coacervation, crowding agents, or LLPS formation; (iv) the ability to quickly and conveniently assay potential effectors and regulators of amyloid-related assembly; and (v) the likely applicability to a wide range of amyloid-forming and other systems. Taken together, the E-CD (Fig. 2) and electrochemically driven DLS (E-DLS) (Fig. 3) results suggest that the higher salt concentration used promotes the more pathological-like, faster, more extensive, and irreversible electroreductive formation of beta-rich and tangled fibrillar assemblies, whereas under the lower-salt condition, the folding and assembly that are observed, particularly after only a short time of electroreduction, may drive more physiologically normal, benign, and reversible changes. The methods we have described may thus facilitate deeper analysis of factors and mechanisms governing transition between normal physiology and amyloidogenic pathology.

In summary, our use of low voltage as a surrogate for phosphorylation has revealed a progressive transition between metastability and irreversibility in charge neutralization–driven folding and assembly leading to beta-rich fibrillar tangles of the K18 domain of tau. Although this transition at the molecular level is revealed, experimentally accessible, and tunably controllable under conditions far from physiological, it is parallel, and may be mechanistically related, to one of the multiple, critical transitions or tipping points, between the prospects for human long-term health, survival, dementia, and death from Alzheimer’s disease and related tau amyloid-associated neurodegenerative pathologies. Extension of the technology described to other spectroscopies offers the prospect of deeper, kinetically resolved analyses of mechanisms governing the early assembly and different trajectories of neutralization-driven amyloid formation by tau and other proteins.

## Experimental procedures

### Proteins

K18-encoding plasmid pNG2-K18 (16) was the generous gift of Prof. Markus Zweckstetter (Göttingen). The protein was expressed in *Escherichia coli*, chromatographically purified, and verified to be pure by SDS-polyacrylamide gel electrophoresis, all as described (16, 67). Assay by Bicinchoninic acid (BCA, Thermo Fisher Scientific) determined the specific extinction coefficient of 1310 M^-1^ cm^-1^. Prior to analysis, samples of lyophilized, purified K18 were (i) dissolved in sodium acetate buffer (25 mM, pH 4); (ii) dialyzed into 1 mN HClO_4_, pH 3, 10 mM or 50 mM NaCl, and diluted with the corresponding medium to 53.5 μM (as measured by *A*_280_); (iii) centrifuged until monomers were the predominant species in solution (as determined by dynamic light scattering, q.v.); and (iv) diluted to a final concentration of 5.35 μM and analyzed at that concentration and under those conditions at 25 °C. Poly-L-lysine (Lys_20_, 3300 kDa) was purchased from Alamanda Polymers and analyzed in aqueous solution of 100 mM NaClO_4_ at pH 3.

### Differential pulse voltammetry

K18 DPV was conducted in 20-μl droplets of analyte (157 μM K18 in 50 mM NaCl pH 3) using a miniaturized three-electrode configuration, with the droplet centered on the horizontal, upwardly facing 3-mm-diameter Pt disc WE, with Pt wire counter electrode (CE), and fritted Ag/AgCl in 1 M KCl(aq) as reference electrode (RE), as described (27). Lys_20_ DPV was conducted in 1.5 ml volume of analyte (2 mM Lys_20_ in 100 mM NaClO_4_ pH 3) in a three-electrode configuration (WE, Pt disk [3 mm]; CE, Pt wire; and RE, fritted Ag/AgCl in 1 M KCl(aq)). Before each measurement, the Pt disc WE was polished three times (2 min each) using 3 μm, 1 μm, 0.25 μm, and then 0.05 μm MetaDiTM polycrystalline diamond suspension (Buehler, Lake Bluff) on a microfiber cloth polishing pad. The polished WE was then sonicated in water for 2 min. DPV measurements were performed with a potential step size of 5 mV, pulse height of 10 mV, pulse duration of 200 ms, and interval time of 0.5 s, with an Autolab M204 electrochemical workstation.

### Electrochemically driven circular dichroism

As recently described (33), CD spectra of K18 (5.35 μM in 1 mN HClO_4_, pH 3, 10 mM and 50 mM NaCl at 25 °C) were measured using a Jasco J-1500 spectropolarimeter with constant N_2_ flushing and temperature-controlled (25 °C) quartz cell (0.2 cm path length) fitted with three electrodes (WE, Pt mesh [geometric area = 8 × 16 mm^2^ of 52 mesh Pt wire of a 100 nm diameter folded in two] in the CD optical beam path; CE, Pt mesh [geometric area = 4 × 8 mm^2^ of 52 mesh Pt wire of a 100 nm diameter folded in two]; and RE, fritted Ag/AgCl in 1 M KCl(aq)). A Gamry electrochemical workstation was used in chronoamperometry mode, permitting simultaneous measurement of ellipticity, absorbance, and photodetector high voltage under voltage bias. CD spectra were recorded between 195 nm and 225 nm with a spectral bandwidth of 1 nm.

### DLS and electrochemically driven DLS

As described (27), E-DLS measurements of K18 (5.35 μM in 1 mN HClO_4_, pH 3, 10 mM and 50 mM NaCl at 25 °C) were conducted with a three-electrode configuration (WE, Pt coil; CE, Pt wire; RE, fritted Ag/AgCl in 1 M KCl(aq)) assembled in a DLS-compatible optical cuvette and sealed with a plug to exclude dust and prevent evaporation, using an Autolab M204 electrochemical workstation (Metrohm). DLS was performed with a Malvern Zetasizer Nano ZS. Samples were probed with a 632.8-nm HeNe gas laser with a beam diameter of 0.63 mm (1/e2) and detected by an avalanche photodiode (quantum efficiency >50% at 633 nm) in a back-scattering configuration at 7° from normal; accuracy of size calibration was verified by analysis of standard silica nanoparticles. Measurements were conducted with 1.25 ml sample volumes at 25 °C at the potentials indicated. K18 solution stability was investigated with no applied voltage over an extended time (11 h) to ensure monomer stability over the time course of the experiments.

### TEM and EDX

After E-CD (Fig. 2), samples were analyzed by TEM and EDX. For TEM, each sample was deposited as a droplet on Parafilm (American National Can), drawn by capillarity through a freshly glow-discharged Formvar-coated copper grid (Electron Microscopy Sciences) that had been floated face-down on the droplet, stained quickly twice with freshly filtered uranyl acetate (2%) by similar capillarity, and imaged at room temperature with a Talos G2 200X, Thermo Fisher Scientific, at the UCSB MRL Shared Microscopy facility. Elemental analyses were conducted by EDX on the same instrument using standard procedures.

### Thioflavin-T fluorescence assay

Presence of beta-structured amyloid fibrils in K18 solution post E-CD was assessed by performing a standard ThT assay (68, 69). Samples of K18 (5.35 uM in 50 mM NaCl 1 mN HCl pH 3) monomer solution (confirmed by DLS) and post E-CD (−0.9 V for 2 h, followed by 40 min at OCP) were diluted to 3 μM in 50 mM Tris buffer, pH 7.4, and mixed with 40 uM of ThT solution. Emission spectra were measured at room temperature between 465 nm and 585 nm on a Horiba FluoroMax 4 fluorescence spectrometer (UCSB’S MRL Shared Experimental Facilities) using an excitation wavelength of 450 nm, normalized to the highest peak at 485 nm.

## Data availability

All data are reported in the article and electronic supporting material.

## Supporting information

This article contains supporting information.

## Conflict of interest

The authors declare that they have no conflicts of interest with the contents of this article.
