# Supplementary material for: A new electrochemical method that mimics phosphorylation of the core tau peptide K18 enables kinetic and structural analysis of intermediates and assembly
Source: J Biol Chem. 2023 Feb 11;299(3):103011. doi: 10.1016/j.jbc.2023.103011 (PMC10024187; doi:10.1016/j.jbc.2023.103011)
Supplement: Supporting information [file mmc1.pdf]

A new electrochemical method that mimics phosphorylation of the core tau peptide K18 enables kinetic and structural analysis of intermediates and assembly

Eloise Masquelier[1,2]*, Esther Taxon[1,3]*, Sheng-Ping Liang[1,4], Yahya Al Sabeh[1,3],
Lior Sepunaru[4], Michael J. Gordon[1,5] and Daniel E. Morse[1,3]**

[1]Institute for Collaborative Biotechnologies
[2]Materials Department; [3]Department of Molecular, Cellular and Developmental Biology;
[4]Department of Chemistry and Biochemistry;[5]Department of Chemical Engineering;
University of California, Santa Barbara, CA 93106 USA
*Contributed equally to this work
**To whom inquiries should be addressed

Supporting Information

The document includes:
- Supporting Figure S1
- Supporting Figure S2
- Supporting Figure S3
- Supporting Figure S4
- Supporting Figure S5
- Supporting Figure S6
- Supporting Figure S7

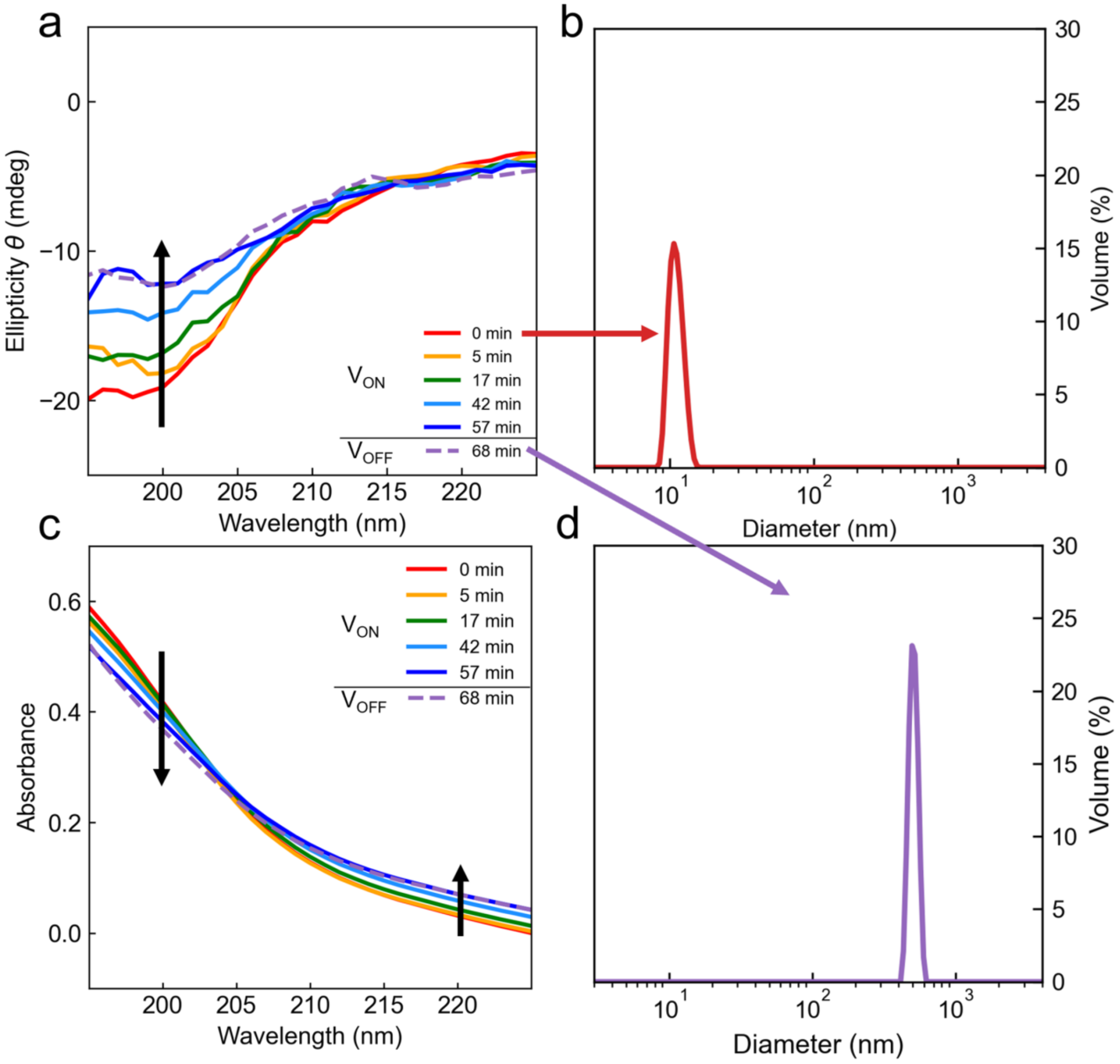

Figure S1. Electroreduction of K18 drives secondary folding and assembly. Dynamic evolution of ellipticity (**a**) and absorbance (**c**) of K18 in 10 mM NaCl, biased at – 0.9 V at time t=0; the potential was held at that level for 60 min and then returned to OCP (V_{OFF}). Black arrows indicate the temporal evolution of CD and absorbance signals during the time the bias is applied. Dynamic light scattering of K18 was analyzed before (t=0) (**b**) and after E-CD (t=69 min) (**d**), revealing a change from monomer to assemblies.

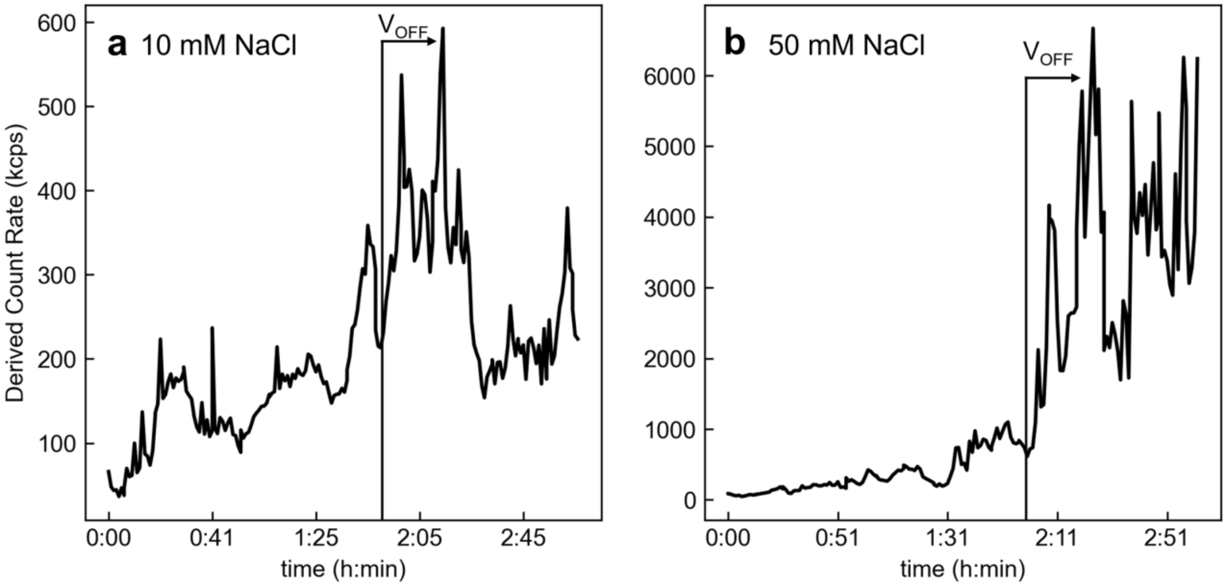

Figure S2. Dynamics of assembly are salt concentration dependent. Derived count-rate of scattering particles of K18 measured by E-DLS at – 0.9 V for 120 min and then followed for an additional 40 min after the potential was returned to OCP, at 10 mM NaCl (**a**) and 50 mM NaCl (**b**). The count-rate, proportional to scattering particle diameter x 10^6, reflects the growth of mass of K18 assemblies.

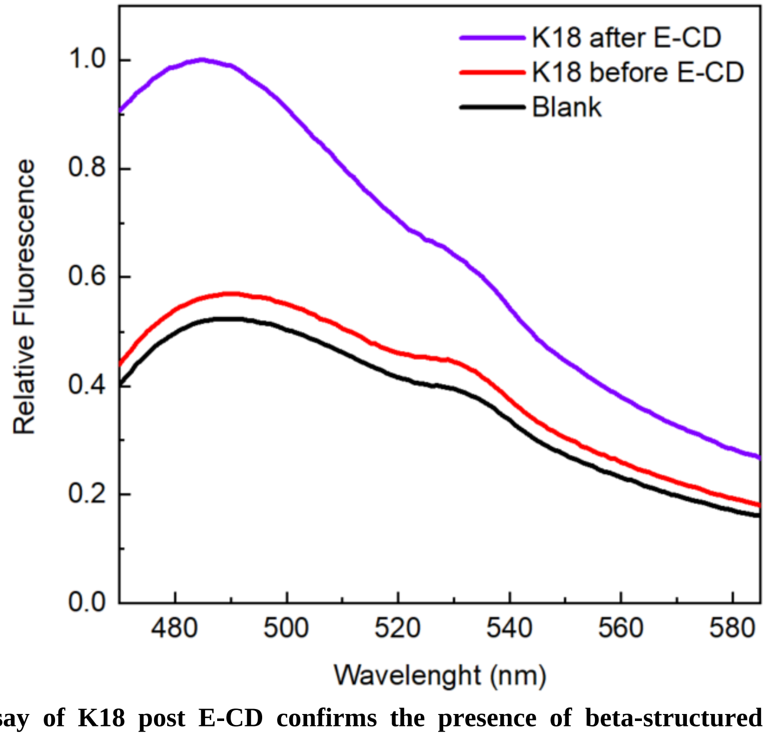

Figure S3. ThT assay of K18 post E-CD confirms the presence of beta-structured fibrils. Normalized fluorescence spectra of K18 after E-CD, K18 before E-CD, and blank solution, excited at 450 nm.

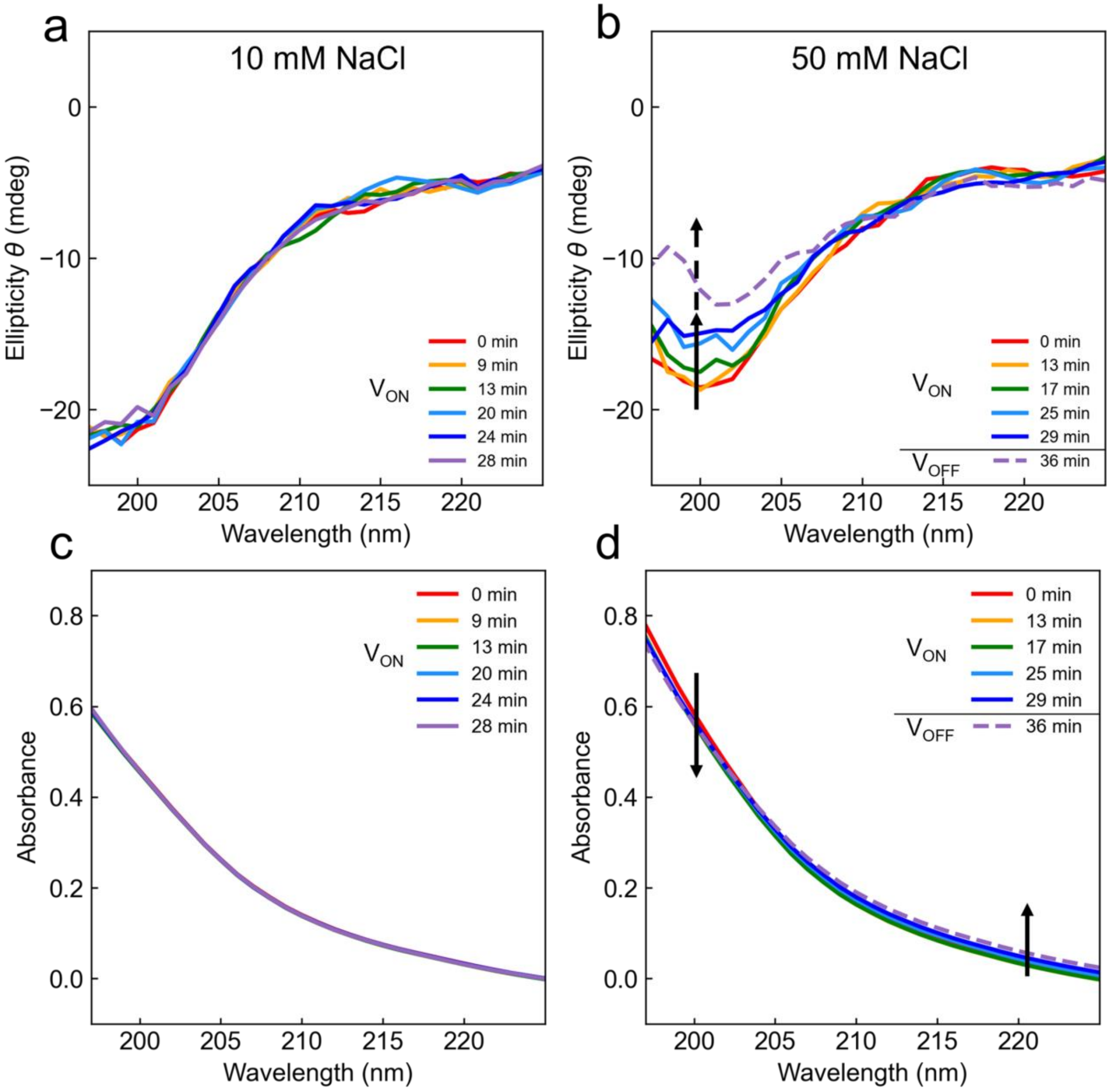

Figure S4. Higher salt concentration facilitates K18 secondary folding at low voltage. Dynamic evolution of ellipticity (**a, b**) and absorbance (**c, d**) of K18 in 10 mM NaCl (**a, c**) and 50 mM NaCl (**b, d**), biased at – 0.45 V. Black arrows indicate the temporal evolution of CD and absorbance signals over time when the bias is applied (V_{ON}, solid) and returned to OCP (V_{OFF}, dashed).

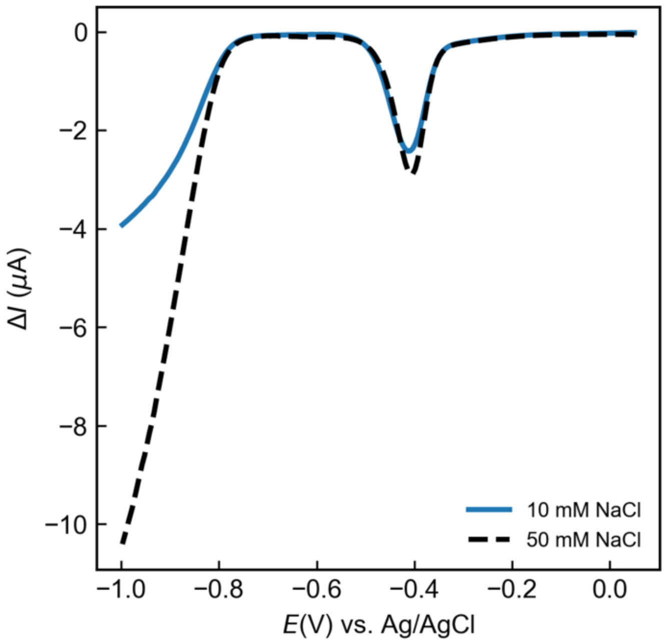

Figure S5. Salt concentration of the blank solutions affects water electrolysis kinetics. Differential pulse voltammograms of the blank solutions (pH 3, 1 mM $HClO_4$) show similar traces of hydronium reduction in both salt concentrations (peak at – 0.4 V vs. Ag/AgCl) but faster kinetics of water electrolysis at 50 mM NaCl (onset at – 0.8 V vs. Ag/AgCl).

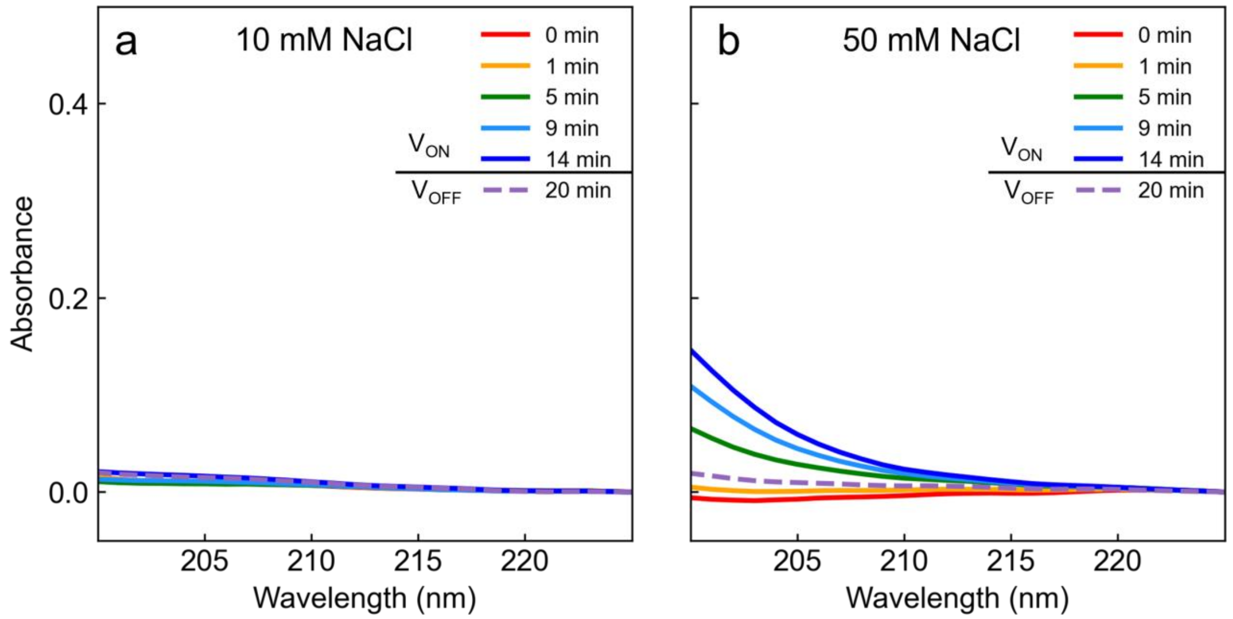

Figure S6. Electroreduction of the blank solution at – 0.9 V leads to a stronger pH gradient in 50 mM NaCl. Dynamic evolution of absorbance as a function of time in 10 mM NaCl (**a**) and 50 mM NaCl (**b**) of the blank solution (1 mM $HClO_4$, pH 3) biased at – 0.9 V vs. Ag/AgCl for 15 min. Absorbance is continuously monitored during and shortly after the bias is returned to OCP (solid curves and dashed curve, respectively). The increase observed in (**b**) is a direct measure of the absorbance of OH^- formed during water electrolysis, followed by a rapid collapse of the gradient when the voltage is switched off.

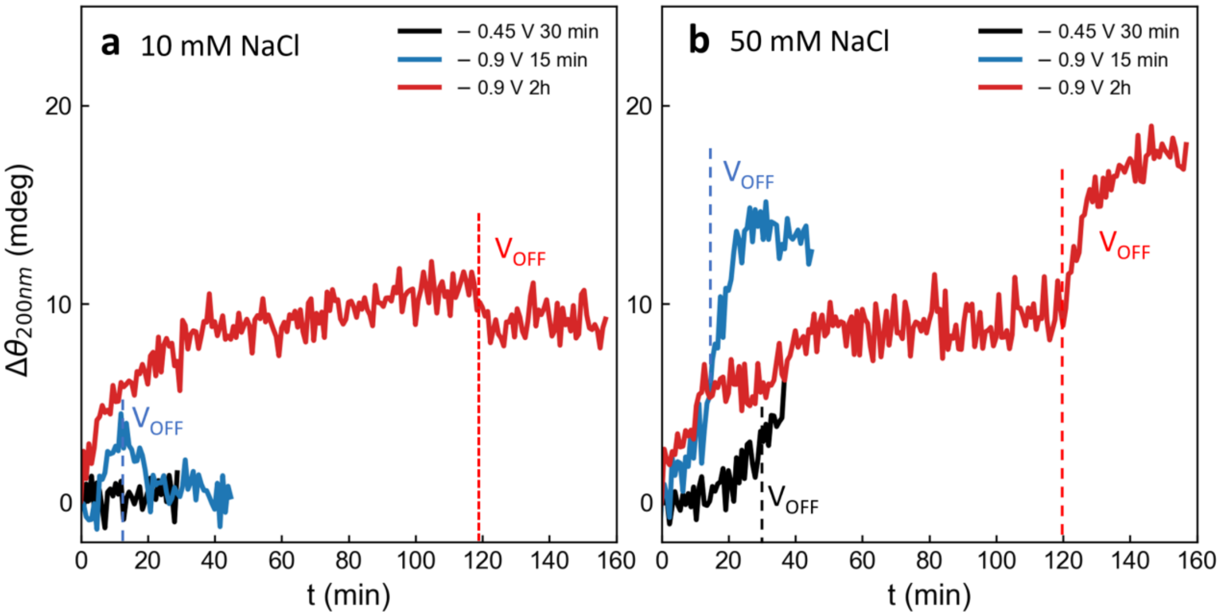

Figure S7. Higher salt concentration promotes continuous structural change after return to OCP. Dynamic evolution of ellipticity monitored at 200 nm as a function of time in 10 mM NaCl (**a)** and 50 mM NaCl (**b**), with bias at – 0.45 V for 30 min (black curves), – 0.9 V for 15 min (blue curves), and – 0.9 V for 2 h (red curves). Ellipticity is continuously monitored during and after the bias is returned to OCP (indicated by the dashed line).
